# Supplementary material for: Neutralization mechanism of a highly potent antibody against Zika virus
Source: Nat Commun. 2016 Nov 24;7:13679. doi: 10.1038/ncomms13679 (PMC5123051; doi:10.1038/ncomms13679)
Supplement: Supplementary Information — Supplementary Figures and Supplementary Tables [file ncomms13679-s1.pdf]

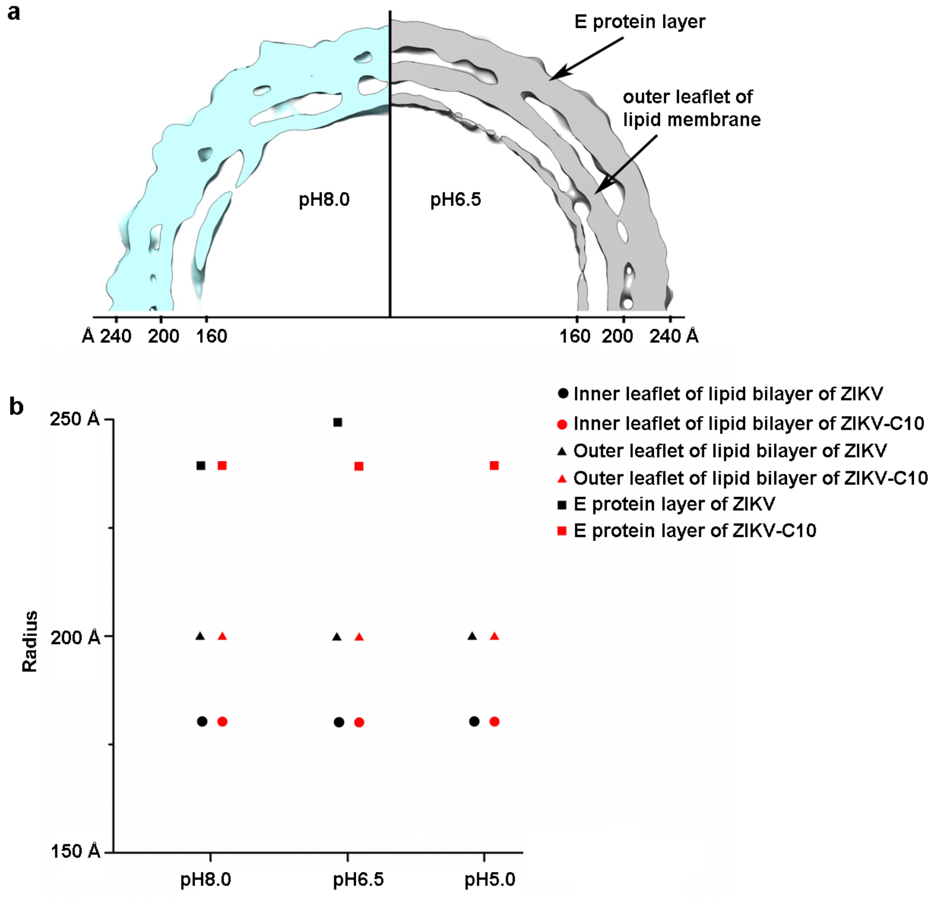

Supplementary Figure 1 | Comparison of the radial positions of the E and the lipid bilayer membrane of ZIKV at pH8.0, pH6.5 and pH5.0. (**a**) Display of a quarter of the central cross-section of the two 25Å resolution cryoEM maps. Compared to the pH8.0 cryoEM map, the E protein layer of the pH6.5 map has a smoother surface, and exists at a slightly higher radius and thus more separated from the outer leaflet of the lipid bilayer membrane. (**b**) Radial distribution of the E protein and the lipid bilayer in the 2D class averages of uncomplexed and complexed ZIKV at pH8.0, pH6.5 and pH5.0. The radius of the E protein layer of averaged particle moved to a higher radius at pH5.0 when the virus bound to C10 compared to the uncomplex control.

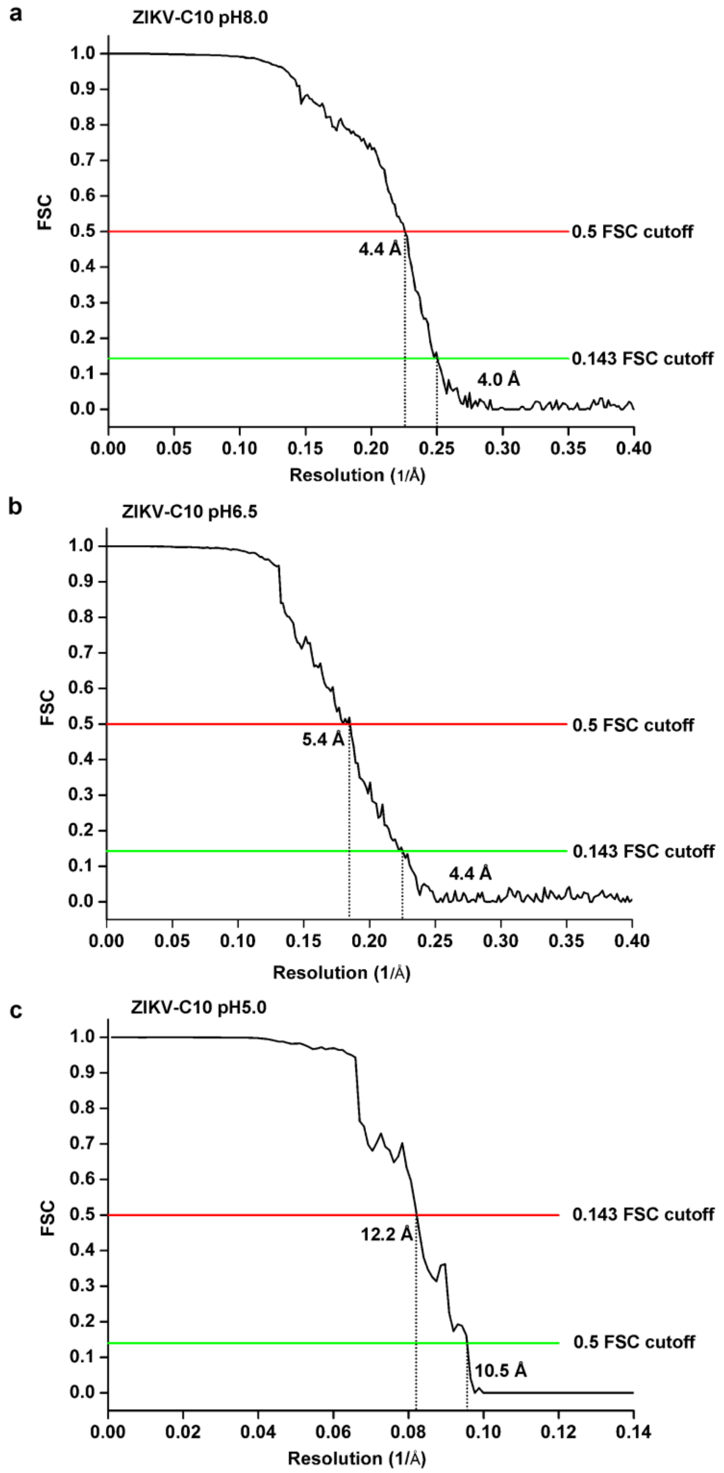

Supplementary Figure 2 | Fourier shell correlation plots for the cryoEM maps of the ZIKV-C10 complexes. For Fab C10-ZIKV complex at pH8.0 and 6.5, we used a 0.143 FSC cutoff to estimate the resolution to be 4.0Å and 4.4Å, respectively. For the Fab C10-ZIKV complex structure

at pH5.0, since it is a medium resolution map, we used a 0.5 FSC cutoff to estimate the resolution to be 12Å.

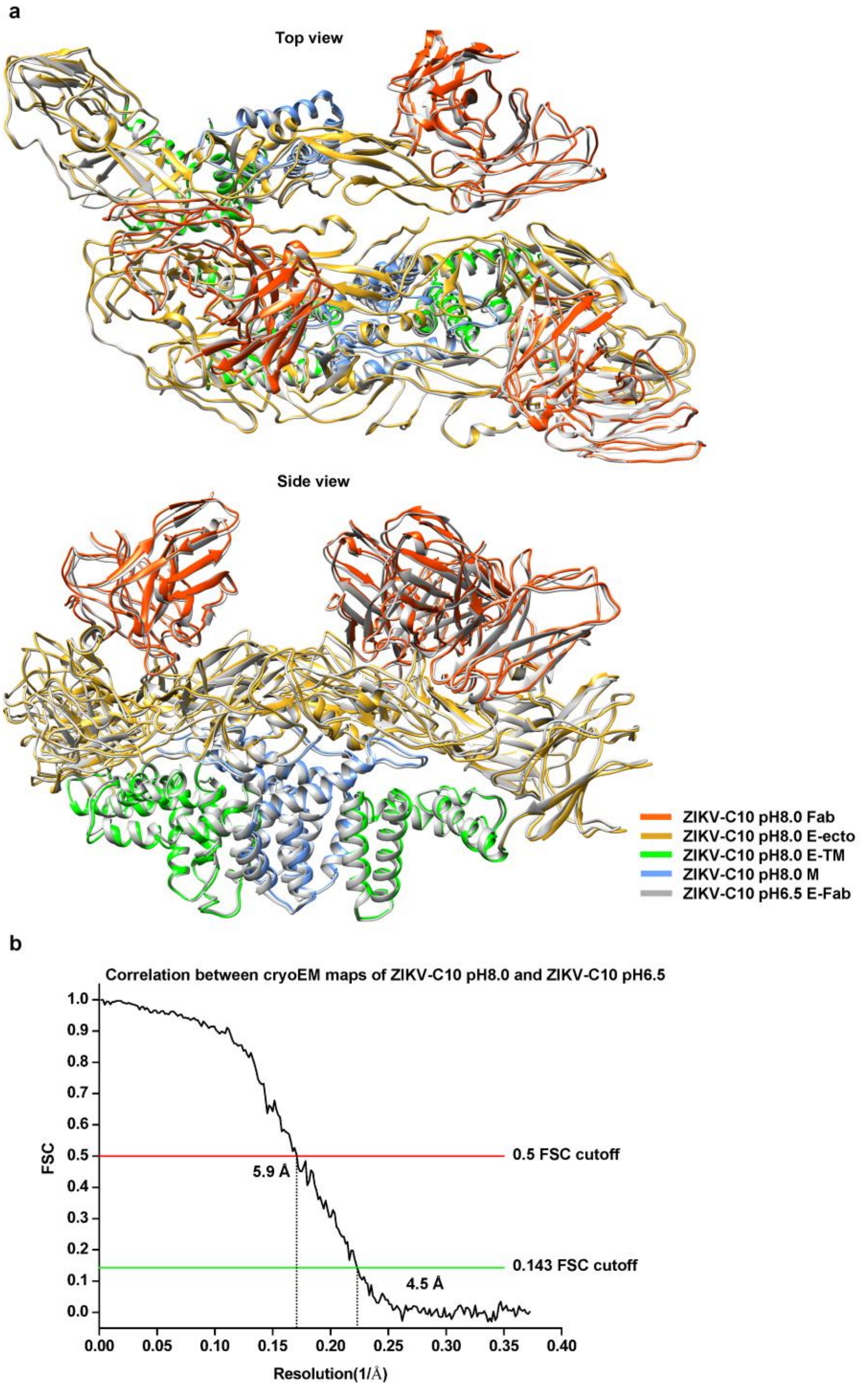

Supplementary Figure 3 | The ZIKV-C10 complex structures at pH8.0 with pH6.5 are highly similar. (**a**) Top and side views of the ribbon representations of the two structures. The RMSD value between these two structures is 0.6Å. (**b**) Fourier shell correlation of the two cryoEM maps, showing the maps are highly similar.

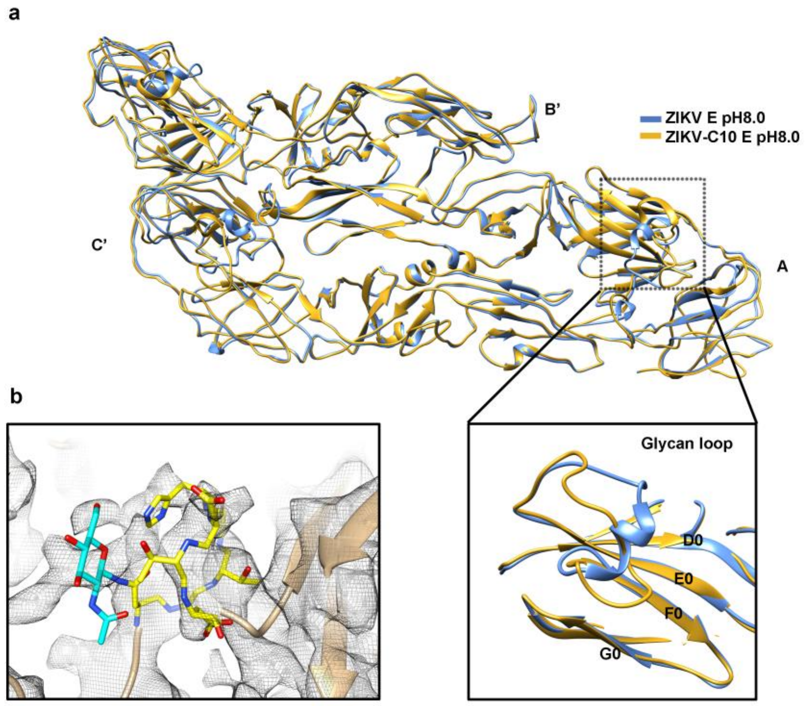

Supplementary Figure 4 | The E protein structures of the uncomplexed ZIKV and the ZIKV-C10 complex at pH8.0 show high similarities except at the "150 glycan loop". (**a**) The ribbon representation of both structures. The "150 glycan loop" changes in conformation when virus is complexed with C10 antibody (inset). E proteins molecules A, B' and C' are indicated. (**b**) Density (grey mesh) of the "150 glycan loop". The Cα backbone of the glycan loop and carbon atoms of the sugar moiety are colored in yellow and cyan, respectively. The nitrogen and oxygen atoms are in red and blue, respectively. The rest of the E protein is shown as light brown ribbon.

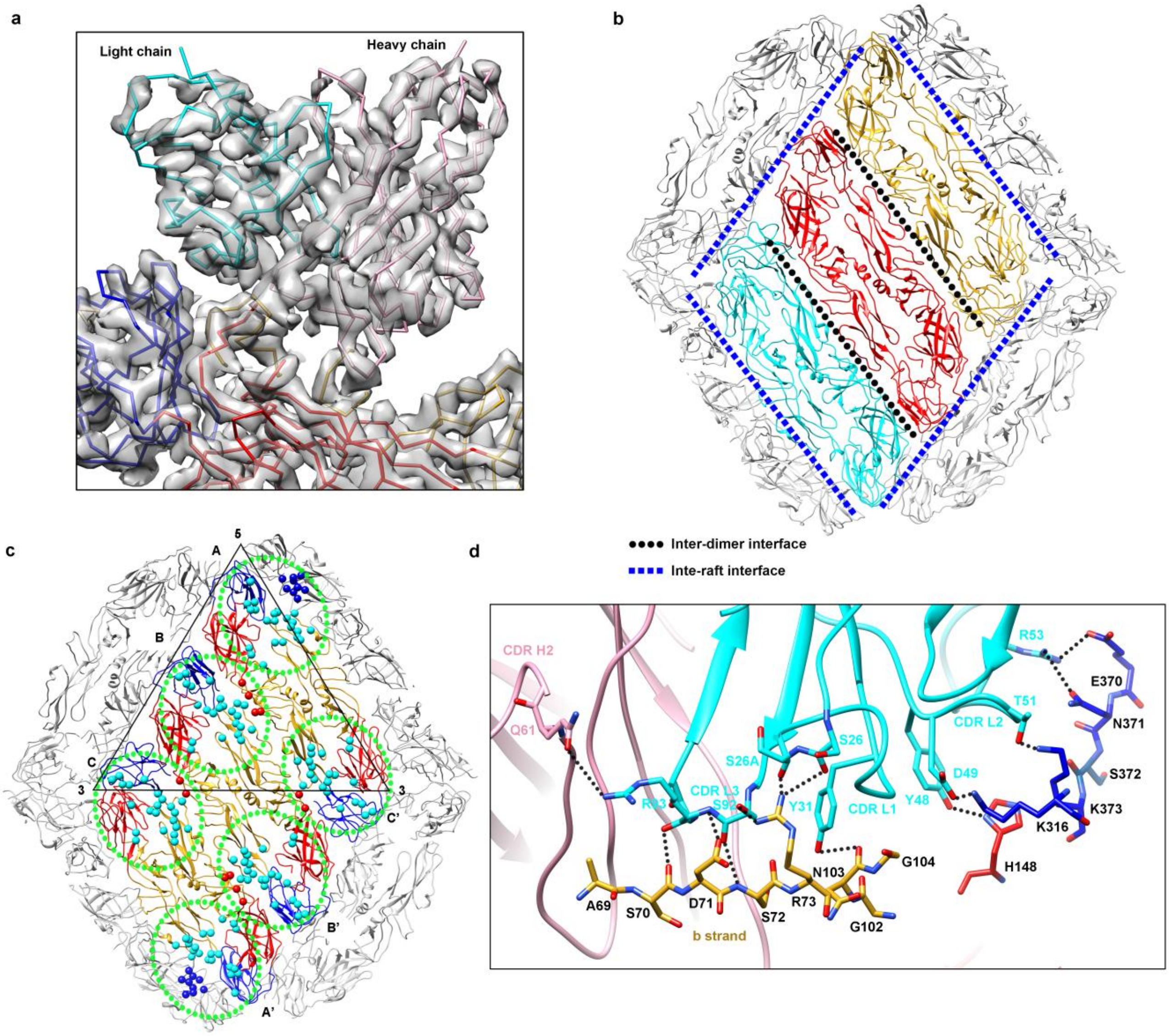

Supplementary Figure 5 | The C10 epitope on an E protein raft identified by different distance cutoff between the Fab and its epitope. (**a**) The fit of C10 Fab into the cryoEM density map of ZIKV-C10 at pH8.0. The variable regions of heavy and light chain are coloured in pink and cyan respectively, whereas DI, DII and DIII of E protein in red, yellow and blue, respectively. (**b**) A schematic diagram showing the inter-dimer (black circle dotted lines) and inter-raft (blue square dotted lines) interfaces. The three E protein dimers in a raft are colored in yellow, red and cyan. Neighboring E proteins in other rafts are colored in grey. (**c**) Boundaries of the C10 epitopes on a

raft identified by a distance cutoff of 8 Å between Cα chains of the Fab and the E protein circled with green dots. Residues forming the intra-dimer epitope are shown as cyan spheres, whereas those at inter-dimer and inter-raft interfaces as red and dark blue spheres, respectively. (**d**) A zoom-in view of part of the interacting interface between C10 Fab and E protein identified using a cutoff of 4 Å distance. Hydrogen bonds and electrostatic interactions are represented as dotted line.

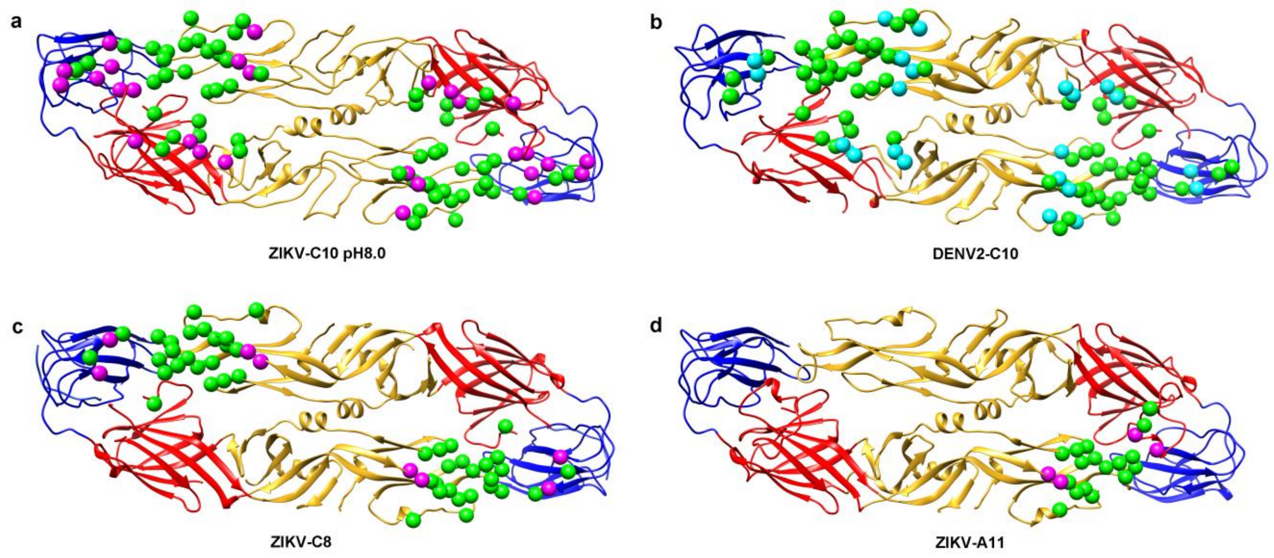

Supplementary Figure 6 | Comparison of the intra-dimer epitope (marked by spheres) of the (a) Fab C10 identified in the ZIKV-C10 pH8.0 cryoEM structure to the previously solved (b) DENV2-C10 crystal structure, and also the (c) C8 and (d) A11 epitopes on ZIKV. Green spheres are the conserved residues (identical or similar in charges and hydrophobicity) between ZIKV and DENV. The unique residues in the epitope on ZIKV and DENV are shown as magenta and cyan spheres, respectively.

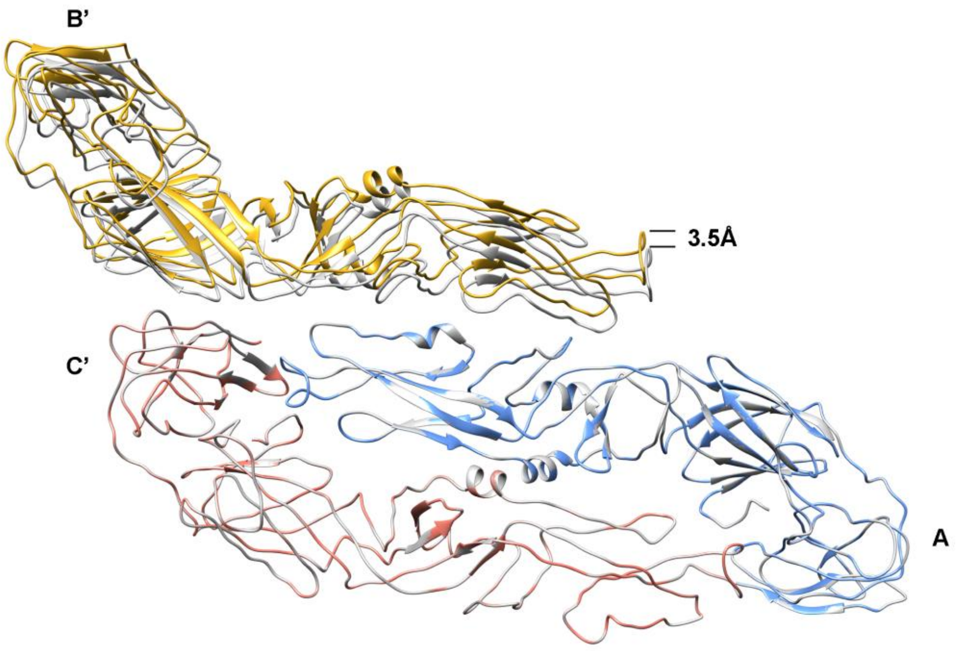

Supplementary Figure 7 | Comparison of the positions of the E proteins in an asymmetric unit of the ZIKV-C10 complex at pH8.0 and pH5.0. The molecule A of the pH8.0 ZIKV-C10 complex structure is aligned with that at pH5.0 while keeping the relative position of the other E proteins fixed. This shows the B’ molecule of the pH5.0 complex structure shifts slightly (~3.5 Å) compared to the pH8.0 structure. This motion does not likely break the interaction of residues on the antibody to its epitope across the interdimer interface (**Supplementary Table 2**). The A, B’ and C’ molecules of the ZIKV-C10 complex structure at pH5.0 are colored in light blue, yellow and salmon, respectively. The E protein molecules of the pH8.0 ZIKV-C10 are colored in light grey.

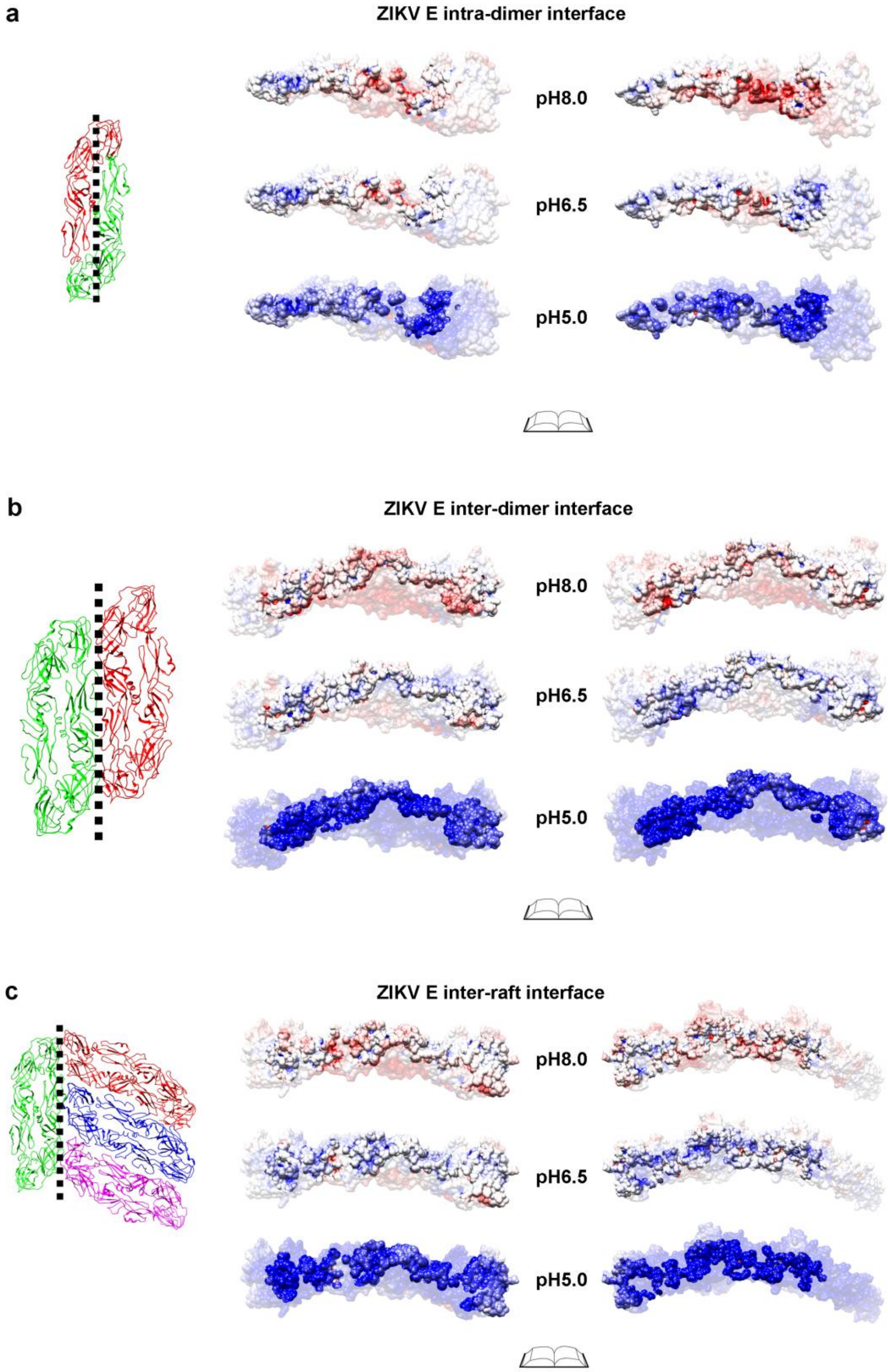
a
ZIKV E intra-dimer interface
pH8.0
pH6.5
pH5.0
b
ZIKV E inter-dimer interface
pH8.0
pH6.5
pH5.0
c
ZIKV E inter-raft interface
pH8.0
pH6.5
pH5.0

Supplementary Figure 8 | Calculated E protein charge distribution at the intra-dimer, inter-dimer and inter-raft interfaces at pH8.0, 6.5 and 5.0. Positive, negative and neutral charge residues are colored in blue, red and white, respectively.

Supplementary Table 1 | List of interaction sites identified by using 5Å distance cutoff between residues of the three individual Fabs C10 with the E proteins in an asymmetric unit in the ZIKV-C10 pH8.0 structure.

C10 Fab			E protein residues<br>Molecules A, B, C in an asymmetric unit indicated as **A**, **B** and **C**, respectively		
**Fab molecule**	Main interacting E protein dimer	Fab residue<br>(**H -** Heavy chain, **L** - light chain, CDR loops and frame work regions are indicated in brackets)	Intra-dimer	Inter-dimer	Inter-raft
**I**	**A-C' (near the 5 fold)**	**H-**G53 (**CDR2**)	**A-**D278		
		**H-**G54 (**CDR2**)	**A-**276**, A-**D278		
		**H-**K58 (**CDR2**)	**C-**A69		
		**H-**Q61 (**CDR2**)	**C-**D67		
		**H-**Q64 (**CDR2**)	**C-**D67		
		**H-**R71 (**FH3**)	**A-**D278		
		**H-**K96 (**CDR3**)	**A-**D155		
		**H-**D98 (**CDR3**)	**A-**R2		
		**H-**D99 (**CDR3**)	**A-**R2,**A-**V46, **A-**M140		
		**H-**Y100 (**CDR3**)	**A-**R2, **A-**H27,**A-**V46,**A-**V47		
		**H-**D100B (**CDR3**)	**C-**K251,		
		**H-**W100D (**CDR3**)	**C-**S70,**C-**K251,**C-**R252, **C-**R253		
		**H-**F100E (**CDR3**)	**C-**S72, **C-**R99,**C-**G102,**C-**N103		
		**H-**P100F (**CDR3**)	**C-**G102		
		**H-**L100H (**CDR3**)	**A-**S149,**A-**G150,**A-**M151		
		**H-**W100I (**CDR3**)	**A-**G150, **A-**V153		
		**L-**S26(**CDR1**)	**C-**R73		**A-**K297
		**L-**G27(**CDR1**)	**C-**Q77		
		**L-**G28(**CDR1**)	**C-**R73,**C-**C74,**C-**Q77		**A-**K297
		**L-**F29(**CDR1**)	**C-**S72, **C-**R73,**C-**C74,**C-**N103		
		**L-**N30(**CDR1**)	**C-**G104		
		**L-**Y31(**CDR1**)	**C-**G102,**C-**N103		
		**L-**Y48(**FL2**)	**A-**G150,**A-**M151, **A-**I152		

		**L**-D49(**CDR2**)	**A**-M151		
		**L**-T51(**CDR2**)	**A**-M151, **A**-N371,**A**-K373		
		**L**-R53(**CDR2**)	**A**-E370,**A**-N371		
		**L**-P54(**CDR2**)	**A**-V153		
		**L**-V57(**FL3**)	**A**-N371		
		**L**-S59(**FL3**)	**A**-E370		
		**L**-K65(**FL3**)			**A**-R299
		**L**-S66(**FL3**)			**A**-L180,**A**-G181,**A**-G182,**A**-G184,**A**-R299
		**L**-G67(**FL3**)			**A**-G184, **A**-R299
		**L**-N68(**FL3**)			**A**-S185
		**L**-S92(**CDR3**)	**C**-S70**,C**-D71,**C**-S72,**C**-R73		
		**L**-R93(**CDR3**)	**C**-A69,**C**-S70,**C**-D71, **C**-L82, **C**-D83, **C**-K84		
		**L**-G94(**CDR3**)	**C**-S70		
**II**	**A-C' (near the 3-fold vertex)**	**H**-K58(**CDR2**)	**A**-M68,**A**-A69, **A**-S70		
		**H**-Q61(**CDR2**)	**A**-K84		
		**H**-D99 (**CDR3**)	**C**-R2,**C**-V46, **C**-R138, **C**-M140,		
		**H**-Y100(**CDR3**)	**A**-H249, **A**-K251, ,**C**-H27,**C**-E44,**C**-V46,**C**-T47, **C**-R283		
		**H**-D100B (**CDR3**)	**A**-R252,**C**-E276		
		**H**-W100D (**CDR3**)	**A**-M68,**A**-S70,**A**-R252, **A**-Q253		
		**H**-F100E (**CDR3**)	**A**-S72, **A**-D98, **A**-N103, **A**-L113,**A**-R252		
		**H**-P100F (**CDR3**)	**A**-W101,**A**-G102		
		**H**-L100H (**CDR3**)	**A**-G102,**A**-N103,**A**-G104,**C**-S149		
		**H**-W100 I(**CDR3**)	**C**-S149		
		**L**-S26(**CDR1**)	**A**-R73	**B'**-E55	
		**L**-S26A (**CDR1**)	**A**-R73		
		**L**-D26B (**CDR1**)	**A**-R73		
		**L**-G27(**CDR1**)	**A**-Q77		
		**L**-G28(**CDR1**)	**A**-C74,**A**-Q77,**A**-C104,**A**-C105		

		**L**-F29(**CDR1**)	**A**-S72,**A**-R73,**A**-C74,**A**-N103,**A**-G104		
		**L**-N30(**CDR1**)	**A**-W101,**A**-N103,**A**-G104,**A**-C105,**A**-G106, **C**-K316		
		**L**-Y31(**CDR1**)	**A**-G102,**A**-N103,**A**-G104		
		**L**-Y48(**FL2**)	**C**-H148,**C**-S149		
		**L**-D49(**CDR2**)	**C**-K316		
		**L**-T51(**CDR2**)	**C**-T315, **C**-N371, **C**-K373		
		**L**-S52(**CDR2**)	**C**-H148,**C**-S372,**C**-K373		
		**L**-R53(**CDR2**)	**C**-T369,**C**-E370, **C**-N371		
		**L**-S59(**FL3**)	**C**-T369,**C**-E370		
		**L**-R60(**FL3**)	**C**-E370		
		**L**-F61(**CDR2**)	**C**-E370		
		**L**-S66(**CDR2**)		**B'**-N52	
		**L**-G67(**CDR2**)		**B'**-N52	
		**L**-S92(**CDR3**)	**A**-S70,**A**-D71,**A**-S72,**A**-R73		
		**L**-R93(**CDR3**)	**A**-S70,**A**-D71,**A**-L82, **A**-D83, **A**-K84		
		**L**-G94(**CDR3**)	**A**-S70		
**III**	**B-B'(near the 2-fold vertex)**	**H**-N54(**CDR2**)	**B**-M277, **B**-R283		
		**H**-K58(**CDR2**)	**B'**-M68, **B'**-A69, **B'**-S70		
		**H**-Q61(**CDR2**)	**B'**-K84		
		**H**-Q64(**CDR2**)	**B'**-D67, **B'**-K84		
		**H**-K96(**CDR3**)	**B**-R164		
		**H**-D98(**CDR3**)	**B**-R2,**B'**-K251		
		**H**-D99(**CDR3**)	**B**-R2, **B**-E44,**B**-V46, **B**-T47, **B**-M140, **B**-R164		
		**H**-Y100 (**CDR3**)	**B**-H27, **B**-G28,**B**-E44,**B**-V46,**B**-T47, **C**-R283		
		**H**-D100B (**CDR3**)	**B'**-R252,**B**-E276,**B**-R283		
		**H**-W100D (**CDR3**)	**B'**-S70, **B'**-T115,**B'**-R252, **B'**- Q253		
		**H**-F100E (**CDR3**)	**B'**-S72, **B'**-R99,**B'**-N103, **B'**-L113, **B'**-R252		

		**H-**P100F (**CDR3**)	**B'**-N103		
		**H-**L100H (**CDR3**)	**B'**-G102,**B-**S149		
		**H-**W100 I(**CDR3**)	**B-**S149		
		**L-**S26(**CDR3**)	**B'**-R73, **B'**-Q77	**A-**A229,**A-**D230	
		**L-**S26A (**CDR1**)	**B'**-R73		
		**L-**G28(**CDR1**)	**B'**-C74,,**B'**-Q77,**B'**-C105		
		**L-**F29(**CDR1**)	**B'**-S72, **B'**-R73,**B'**-C74, **B'**-R99,**B'**-N103,**B'**-G104,**B'**-C105		
		**L-**N30(**CDR1**)	**B'**-G104,**B'**-C105,**B'**-G106, **B-**K316		
		**L-**Y31(**CDR1**)	**B'**-N103,**B'**-G104		
		**L-**Y48(**FL2**)	**B-**H148, **B-**S149		
		**L-**D49(**CDR2**)	**B-**S149, **B-**K316		
		**L-**T51(**CDR2**)	**B-**T315, **B-**E329, **B-**Q331, **B-**K373		
		**L-**S52(**CDR2**)	**B-**148,**B-**T315, **B-**E329, **B-**Q331,**B-**K373		
		**L-**R53(**CDR2**)	**B-**148, **B-**E370, **B-**N371, **B-**K373		
		**L-**V57(**FL3**)	**B-**T369		
		**L-**T59(**FL3**)	**B-**T369,**B-**E370		
		**L-**S66(**FL3**)		**A-**N52	
		**L-**G67(**FL3**)		**A-**N52	
		**L-**S92(**CDR3**)	**B'**-S70,**B'**-D71,**B'**-S72, **B'**-R73		
		**L-**R93(**CDR3**)	**B'**-A69,**B'**-S70,**B'**-D71,**B'**-S72, **B'**-L82, **B'**-D83, **B'**-K84		
		**L-**G94(**CDR3**)	**B'**-D71		

Supplementary Table 2 | The distances between the interacting residues of Fab C10 and the E proteins at the inter-dimer interface of the ZIKV-C10 complex structure at pH8.0 and pH5.0.

**Interacting residues**		**Distances between Cα chains of the interacting Fab and E protein at the interdimer interface.**	
**C10 Fab**<br>**H-** heavy chain<br>**L**-light Chain	**ZIKV E protein**<br>Molecules A, B and C are indicated as **A**, **B** and **C,** respectively.	**ZIKV-C10 pH8.0 structure**	**ZIKV-C10 pH5.0 structure**
**L**-G67	**B'-**N52	6.3 Å	6.5 Å
**L**-S26	**B'-**A54	7.6 Å	8.0 Å
**L**-G28	**B'-**A54	8.0 Å	9.6 Å
**H**-G67	**A**-N52	6.1 Å	6.9 Å
**H**-S26	**A**-A54	7.9 Å	8.9 Å
**H**-S26	**A**-A229	7.4 Å	7.6 Å
